# Combined Effect of Sarcopenia and Impaired Respiratory Function on All‐Cause Death: A Nationwide Cohort Study

**DOI:** 10.1002/jcsm.70275

**Published:** 2026-04-05

**Authors:** Chutao Wu, Dan Wu, Mu Li, Senrong Lu, Yukai Wang, Yulong Lan

**Affiliations:** ^1^ Department of Emergency Shantou Central Hospital Shantou Guangdong China; ^2^ Shantou University Medical College Shantou Guangdong China; ^3^ Second Affiliated Hospital of Shantou University Medical College Shantou Guangdong China; ^4^ Centre for Precision Health Edith Cowan University School of Medical and Health Sciences Perth Joondalup Australia; ^5^ Department of Neurosurgery Second Affiliated Hospital of Shantou University Medical College Shantou Guangdong China; ^6^ Department of Rheumatology and Immunology Shantou Central Hospital Shantou Guangdong China; ^7^ Department of Cardiology Second Affiliated Hospital of Shantou University Medical College Shantou Guangdong China

**Keywords:** all‐cause death, cohort study, peak expiratory flow, respiratory function, sarcopenia

## Abstract

**Background:**

Respiratory fitness and sarcopenia status have been reported to be cross‐sectionally linked with each other and increase mortality risk. However, little is currently known regarding the connection between impaired respiratory function and sarcopenia and their joint effect on future death risk.

**Methods:**

This study included 12 027 participants (50.8% were women; mean [SD] age, 58.9 [9.3] years) from a nationwide, prospective cohort in China (China Health and Retirement Longitudinal Study). Respiratory function was assessed by peak expiratory flow (PEF). Sarcopenia status was assessed according to the Asian Working Group for Sarcopenia 2019 (AWGS 2019) criteria. Time‐to‐event survival analyses and causal mediation analyses were conducted to assess the joint association of sarcopenia and reduced PEF with all‐cause mortality.

**Results:**

During a mean follow‐up of 8.64 years, 1536 deaths were recorded. After multivariable adjustment, the hazard ratios (HRs) for mortality were 1.51 (95% CI: 1.33–1.71) for possible sarcopenia and 1.67 (95% CI: 1.43–1.94) for diagnosed sarcopenia. A dose‐dependent association between PEF and mortality was observed (*p*‐nonlinearity > 0.05). The adjusted HRs per 1‐SD decrease in PEF were 1.29 (95% CI: 1.18–1.41), 1.15 (95% CI: 1.03–1.29), and 1.49 (95% CI: 1.30–1.71) among individuals with nonsarcopenia, possible sarcopenia, and diagnosed sarcopenia, respectively (*p* interaction = 0.127). Causal mediation analysis demonstrated a bidirectional mediation effect, with both natural direct and indirect effects being statistically significant. Diagnosed sarcopenia was associated with excess mortality partly mediated by reduced PEF (total effect HR: 1.63, 95% CI: 1.38–1.92; natural indirect effect HR: 1.10, 95% CI: 1.07–1.14; proportion mediated: 18.5%). Conversely, sarcopenia mediated 10.0% and 7.0% of the reduced PEF–mortality pathway for possible and diagnosed sarcopenia, respectively. Compared with participants who had normal PEF and no sarcopenia, the adjusted HRs (95% CIs) for mortality were 1.52 (1.22–1.89) for possible sarcopenia with normal PEF, 1.73 (1.28–2.35) for diagnosed sarcopenia with normal PEF, 1.60 (1.36–1.88) for impaired PEF without sarcopenia, 2.22 (1.87–2.64) for impaired PEF with possible sarcopenia, and 2.44 (2.01–2.97) for impaired PEF with diagnosed sarcopenia. No significant risk heterogeneity was observed across sex, age or lifestyle subgroups.

**Conclusions:**

Sarcopenia and impaired respiratory function are interrelated and jointly elevate mortality risk in middle‐aged and older Chinese adults.

## 1 Introduction

Sarcopenia, characterized by age‐related loss of muscle mass, strength, and function, is now recognized as a significant geriatric syndrome affecting approximately 10% of old adults—a figure projected to rise substantially [1]. A meta‐analysis estimated the global prevalence of sarcopenia among elderly individuals to range from 10% to 16%, with variations depending on the study setting and definition [2]. In Europe, the number of sarcopenic patients is expected to increase by 72% by 2045, which will substantially increase the public health burden and negatively affect quality of life [3]. In China, data‐based meta‐analyses have reported an overall prevalence of approximately 11%–13% among community‐dwelling older adults [4, 5]. Sarcopenia has been strongly linked to adverse health outcomes, including cardiovascular disease [6], frailty [7], reduced immune function [8] and increased susceptibility to infections [9, 10] as well as mortality in both the general population [11] and patient cohorts [12, 13, 14]. Despite growing research, the mechanisms underlying its strong association with mortality remain poorly understood.

Impaired lung function, an additional key clinical indicator of mortality risk for a wide range of morbidities [15], has been increasingly linked with sarcopenia. Converging evidence suggests a strong cross‐sectional relationship between sarcopenia and respiratory function. Data from cross‐sectional studies demonstrated a higher prevalence of limb muscle dysfunction [16] and sarcopenia [17, 18] among individuals with poor lung function. For example, a meta‐analysis based on 10 articles involving 2565 chronic obstructive pulmonary disease (COPD) patients demonstrated that among individuals with COPD, the prevalence of sarcopenia was 21.6% and was much higher (63%) in those living in nursing homes [18]. Additionally, sarcopenia is significantly associated with deterioration of respiratory force generation [19] and pulmonary function [20]. Sarcopenic elderly individuals exhibit significantly lower maximal respiratory pressures, with respiratory muscle strength inversely related to sarcopenia and its indicators [19]. A positive association between pulmonary function and muscle strength/physical performance also underscores the importance of considering the interplay between sarcopenia and respiratory function in the pathophysiology of adverse health outcomes [20].

Given the strong association between sarcopenia and impaired respiratory function, these conditions likely interact and contribute to reduced survival in the general population. However, limited research has examined their combined impact on long‐term mortality risk or how lifestyle factors may modify this relationship. To address this gap and improve strategies for precise risk assessment and early targeted interventions, we conducted a study using data from a nationwide cohort in China to investigate the joint effect of sarcopenia and impaired respiratory function on all‐cause mortality in Chinese adults.

## 2 Design and Methods

### 2.1 Study Population

This study is a secondary analysis of data from the China Health and Retirement Longitudinal Study (CHARLS), a nationally representative cohort designed to assess the health, economic, and social conditions of middle‐aged and older adults in China [21]. The CHARLS study was approved by the Biomedical Ethics Review Committee of Peking University (IRB00001052–11015), with informed consent obtained from all participants. Detailed descriptions of the study have been previously published [6, 22]. In brief, the CHARLS was initiated in 2011; 17 708 participants aged 45 years or older were recruited through multistage probability sampling from 450 communities in China. For this analysis, baseline data from the first wave of the health survey from 2011 to 2012 were used. Participants were excluded if they (1) had incomplete information on age, sex or age < 45 years (*n* = 846); (2) lacked data on height, body weight, peak expiratory flow (PEF), or variables for assessing sarcopenia (handgrip strength, five‐time chair stand test, or habitual gait speed) (*n* = 4831) or (3) died before follow‐up (*n* = 4). A total of 12 027 participants were eligible for this study. The participant recruitment flowchart is shown in the Supplementary Figure S1. This present study adheres to the Strengthening the Reporting of Observational Studies in Epidemiology (STROBE) guidelines [23].

### 2.2 Exposure

Sarcopenia was assessed via the Asian Working Group for Sarcopenia (AWGS) 2019 criteria [24], which integrate muscle strength, appendicular skeletal muscle mass (ASM) and physical performance. Diagnosed sarcopenia was diagnosed when low muscle mass was combined with either low muscle strength or low physical performance. Possible sarcopenia was defined as the presence of low muscle strength or low physical performance alone. Low muscle strength was defined as a grip strength < 28 kg for men and < 18 kg for women. Low physical performance was defined as a habitual gait speed < 1.0 m/s or a five‐time chair rise time ≥ 12 s. Handgrip strength was measured via a Yuejian WL‐1000 dynamometer (Nantong Yuejian Physical Measurement Instrument Co. Ltd., Nantong, China) in both the dominant and nondominant hands. For habitual gait speed, each participant was asked to walk a 2.5‐m course at a usual pace twice (there and back), as previously described [6]. The five‐time chair rise test assessed the time required for participants to rise from a 47‐cm chair five times consecutively. ASM was estimated via a validated anthropometric equation for Chinese residents, which has been shown to be consistent with dual x‐ray absorptiometry (DXA) [6, 22]. Estimation of

$$\begin{array}{c} \mathrm{ASM}=0.193\times \text{weight}\;\left(\mathrm{kg}\right)+0.107\times \text{height}\;\left(\mathrm{cm}\right)-4.157\\ \;\times \text{gender}\;\left(\text{male}:1;\text{female}:2\right)-0.037\times \mathrm{age}\left(\text{years}\right)-2.637 \end{array}$$

As previously suggested [6], low muscle mass was defined on the basis of the sex‐specific lowest 20% of height‐adjusted ASM (ASM/height^2^) in the study population. In this cohort, low muscle mass was defined as an ASM/height^2^ < 5.29 kg/m^2^ for women and < 7.01 kg/m^2^ for men, which is consistent with previous findings in the same cohort [6]. Respiratory function was assessed by PEF, defined as the maximum flow rate achieved during a forced expiratory manoeuvre. The PEF was measured via a vital peak flow meter (Everpure, Shanghai, China), with the results recorded only if maximal effort was demonstrated. Details of exposure assessment are provided in **Supplementary eMethods**. In the analysis, respiratory dysfunction was defined as a per‐standard deviation (SD) decrease in PEF (continuous form) and by the COPD Assessment in Primary Care to Identify Undiagnosed Respiratory Disease and Exacerbation Risk (CAPTURE) criteria, with impaired PEF defined as < 250 L/min for females and < 350 L/min for males [25], which has also been adopted in the Chinese population [26].

### 2.3 Outcome

The primary outcome was all‐cause mortality. Death data were available up to the last CHARLS survey [27], with person‐time calculated from the date of enrolment to the date of death or the last visit before December 2020, whichever occurred first. From April 2011 to December 2020, all deaths were recorded. Mortality data were collected through life history surveys conducted in 2013, 2014, 2015, 2018 and 2020, with a median follow‐up period of approximately 9 years. Participant survival status was verified through field investigations, where interviewers visited participants' residences. The death information was collected by interviewing household members who lived with the deceased.

### 2.4 Covariates

Covariates in this study included demographics (age, sex, residence and education attainment), anthropometrics (height, body weight and blood pressure), lifestyle factors (smoking, alcohol consumption and physical activity) and health‐related information (prevailing cardiovascular disease, hypertension, diabetes, dyslipidaemia, arthritis and cancer). Data were collected by trained interviewers via standardized questionnaires aligned with leading international aging studies, such as the Health and Retirement Study (HRS), the English Longitudinal Study of Aging (ELSA) and the Survey of Health, Aging, and Retirement in Europe (SHARE). Comprehensive quality control procedures were implemented during data collection and verification to ensure the reliability and accuracy of the CHARLS data, facilitating meaningful cross‐population comparisons and analyses (http://charls.pku.edu.cn/). Body mass index (BMI) was calculated by dividing measured weight (kg) by height squared (m^2^). Physical activity was categorized as either active or inactive, with ‘active’ defined as engaging in moderate‐intensity physical activity ≥ 6 times/week or vigorous‐intensity physical activity ≥ 3 times/week. Smoking habits were categorized as never‐smokers, former smokers, or current smokers. Alcohol consumption was categorized as current drinkers or nondrinkers.

### 2.5 Statistical Analysis

The data on the covariates were almost complete. For the description of baseline characteristics, continuous variables with a normal distribution are presented as the means and SD, and categorical variables are presented as frequencies and percentages. Differences in baseline information across different respiratory functions were compared via the chi‐square test for categorical variables and an unpaired Student's *t*‐test or Mann–Whitney *U* test for continuous variables, as appropriate.

The absolute mortality risk was presented as unadjusted incidence rates (per 1000 person‐years) and Kaplan–Meier failure functions. After confirming the satisfaction of the proportional hazard assumption by plotting the log(−log (survival)) versus log (survival time) and by using Schoenfeld residuals, Cox proportional hazards models were employed to estimate hazard ratios (HRs) and 95% confidence intervals (CIs) for mortality associated with sarcopenia and impaired PEF, both individually and in combination. The multivariable‐adjusted models were as follows: Model 1: adjusted for sex, age, residence, education attainment, smoking habits, alcohol consumption, physical activity and disease status (diabetes, hypertension, cardiovascular disease, arthritis, dyslipidaemia and cancer); and Model 2: additionally adjusted for BMI. To test the linear relationship, the dose–response associations between PEF and death incidence in the overall population and across different sarcopenia strata were tested via restricted cubic spline (RCS) curves based on multivariable Cox regression models, with three knots placed at the 25th, 50th and 75th percentiles.

To examine the joint effect of sarcopenia and impaired PEF on mortality, we performed both interaction and mediation analyses. Interaction effects were assessed on multiplicative scales. The multiplicative interaction (INTm) was evaluated via likelihood ratio tests. Causal mediation analysis was conducted using the SAS %MEDIATE macro based on VanderWeele's counterfactual framework [28], which has been widely used for causal inference in survival analysis. We assessed whether reduced PEF (per SD decrease) mediated the association between sarcopenia status and mortality, and conversely, whether sarcopenia status mediated the excess mortality risk of reduced PEF. All models were adjusted for potential confounders, and effect estimates were expressed as HRs with 95% CIs.

To explore risk heterogeneity across different demographic factors and lifestyle patterns, we examined the interaction effects of combined sarcopenia and impaired PEF exposure with covariates on mortality risk by including two‐factor interaction terms in the Cox proportional hazards model. To assess the robustness of our findings, we conducted several sensitivity analyses, excluding participants who died within the first year of follow‐up, those with lung diseases or asthma that could confound the results, and those with arthritis that may contribute to physical inactivity and sarcopenia. Additionally, we redefined impaired PEF according to the Chinese criteria established by Nanshan Zhong et al., where impaired PEF is defined as a PEF < 80% of the predicted value (pPEF) [29]. The sex‐specific algorithms for calculating pPEF in the Chinese population are provided in the **Supplementary Methods**.

All the statistical analyses were conducted via SAS software (Version 9.4; SAS Institute, Cary, NC) and R software (Version 4.5.0). A two‐tailed *P* value < 0.05 was considered statistically significant, except for the multiplicative interaction tests.

## 3 Results

The characteristics of the overall study population and across different respiratory functions are presented in Table 1. The mean age of the study population was 58.9 years (SD: 9.3), with 48.2% being male. Among middle‐aged and older Chinese adults, the prevalence of impaired respiratory function (CAPTURE‐defined impaired PEF) was 47.6%, while the prevalence of possible and diagnosed sarcopenia was 25.0% and 11.3%, respectively. Compared with individuals with normal PEF, those with impaired PEF were older, had a lower BMI and lower educational attainment and were more likely to be former or current smokers and physically inactive. The prevalence of possible sarcopenia (29.7% vs. 20.6%) and diagnosed sarcopenia (18.4% vs. 4.8%) was significantly higher in individuals with impaired PEF. In addition, lung disease, asthma, cardiovascular disease, arthritis, diabetes, dyslipidaemia, hypertension and kidney disease were more common in the impaired PEF group.

**TABLE 1:** Baseline characteristics of the study population.

	Total (*N* = 12 027)	Normal PEF (*n* = 6297)	Impaired PEF (*n* = 5730)	*p‐*difference
Death cases, no. (%)	1536 (12.8)	430 (6.8)	1106 (19.3)	< 0.001
Survival time, mean (SD), years	8.64 ± 1.38	8.83 ± 1.00	8.44 ± 1.68	< 0.001
Age, mean (SD), years	58.94 ± 9.31	56.12 ± 7.92	62.04 ± 9.72	< 0.001
Male, no. (%)	5801 (48.2)	3034 (48.2)	2767 (48.3)	0.920
Sarcopenia status, no. (%)				< 0.001
Possible sarcopenia	3002 (25.0)	1229 (20.6)	1701 (29.7)	
Diagnosed sarcopenia	1358 (11.3)	302 (4.8)	1056 (18.4)	
BMI, mean (SD), kg/m^2^	23.49 ± 3.92	24.03 ± 3.86	22.90 ± 3.89	< 0.001
Residing in urban areas	4441(36.9)	2423 (38.5)	2018 (35.2)	< 0.001
Education, no. (%)				< 0.001
Below lower secondary	10 761 (89.5)	5432 (86.3)	5329 (93.0)	
Upper secondary and vocational training	1095 (9.1)	744 (11.8)	351 (6.1)	
Tertiary education	171 (1.4)	121 (1.9)	50 (0.9)	
Current drinkers, no. (%)	4019 (33.4)	2178 (34.6)	1841 (32.1)	0.005
Smoking habits, no. (%)				< 0.001
Never‐smokers	7202 (58.9)	3862 (61.3)	3340 (58.3)	
Former smokers	1060 (8.8)	506 (8.0)	554 (9.7)	
Current smokers	3765 (31.3)	1929 (30.6)	1836 (32.0)	
Physically active, no. (%)	2899 (24.1)	1580 (25.1)	1319 (23.0)	0.008
Hypertension, no. (%)	4955 (41.2)	2349 (37.3)	2606 (45.5)	< 0.001
CVD, no. (%)	1810 (15.1)	778 (12.4)	1032 (18.0)	< 0.001
Diabetes, no. (%)	1471 (12.2)	732 (11.6)	739 (12.9)	0.036
Dyslipidemia, no. (%)	1234 (10.3)	691 (11.0)	543 (9.5)	0.008
Arthritis, no. (%)	4405 (36.6)	2600 (33.9)	1805 (41.3)	< 0.001
Kidney disease, no. (%)	1038 (8.6)	476 (7.6)	562 (9.8)	< 0.001
Lung disease, no. (%)	1435 (11.9)	423 (6.7)	1012 (17.7)	< 0.001
Asthma, no. (%)	538 (4.5)	104 (1.7)	434 (7.6)	< 0.001
Cancer, no. (%)	127 (1.1)	65 (1.0)	62 (1.1)	0.859

Abbreviations: BMI, body mass index; CVD, cardiovascular disease; No., number; PEF, peak expiratory flow; SD, standard deviation.

**FIGURE 1:**
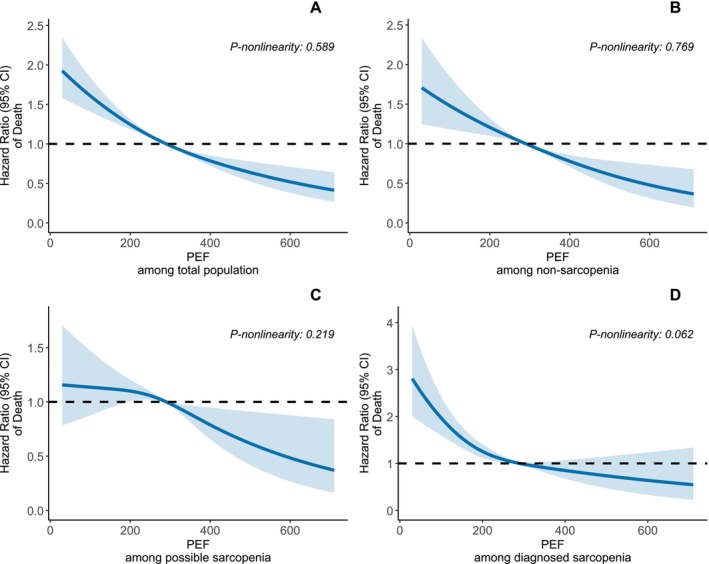
Multivariable dose–response associations between peak expiratory flow (PEF) and all‐cause mortality in the overall population and across sarcopenia and sex subgroups Panel **A** shows results for the overall population; Panel B shows results for nonsarcopenic individuals; Panel **C** shows results for individuals with possible sarcopenia; Panel **D** shows results for individuals with diagnosed sarcopenia. Multivariable Cox models were adjusted for sex (male or female), age (continuous), body mass index (continuous), residence (urban or rural), education level (below lower secondary, upper secondary and vocational training, or tertiary education), smoking status (never, ever, or current), alcohol consumption (yes or no), physical activity (active or inactive) and comorbidities (diabetes, hypertension, cardiovascular disease, arthritis, dyslipidemia, and cancer).

**FIGURE 2:**
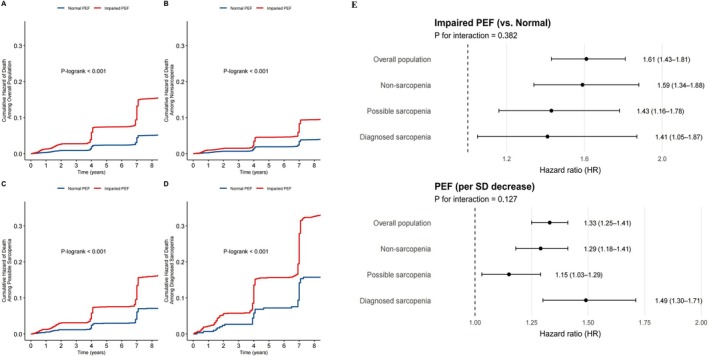
Association between peak efflux flow (PEF) and all‐cause mortality in overall population and across sarcopenia subgroups Panels **A**–**D** show Kaplan–Meier curves of cumulative hazards of death by PEF status (normal vs. impaired) in the overall population (**A**), nonsarcopenia (**B**), possible sarcopenia (**C**) and diagnosed sarcopenia (**D**). Panel **E** presents hazard ratios (HRs) for impaired vs. normal PEF and per–SD decrease in PEF from multivariable‐adjusted Cox models, stratified by sarcopenia status. *P* values for interaction were calculated to test effect modification by sarcopenia in the multivariable model. Covariates in the multivariable model includes sex (male or female), age (continuous), body mass index (continuous), residence (urban or rural), education level (below lower secondary, upper secondary and vocational training, or tertiary education), smoking habits (never, ever or current), alcohol consumption (yes or no), physical activity (active or inactive) and disease status (diabetes, hypertension, cardiovascular disease, arthritis, dyslipidemia and cancer).

During a mean follow‐up of 8.64 years (SD: 1.38), 1536 deaths were recorded. As shown in Table 2, mortality risk increased progressively with worsening sarcopenia status (*p*‐trend < 0.001). The incidence rates of death were 9.29, 18.06 and 40.99 per 1000 person‐years in the nonsarcopenia, possible sarcopenia, and diagnosed sarcopenia groups, respectively. After adjustment for sociodemographic characteristics, lifestyle factors, comorbidities and medication use, the risks were 1.44 (95% CI: 1.28–1.64) for possible sarcopenia and 1.93 (95% CI: 1.68–2.22) for diagnosed sarcopenia. Additional adjustment for BMI slightly attenuated the associations (HR: 1.51, 95% CI: 1.33–1.71; HR: 1.67, 95% CI: 1.43–1.94, respectively). The RCS curves (Figure 1 and Supplementary eFig. S2) demonstrated broadly consistent dose–dependent associations between reduced PEF and mortality in the overall population and across both sexes, with slightly steeper slopes observed in women, particularly at lower ranges of PEF. Except for men with possible sarcopenia (*p*‐nonlinearity = 0.023), the associations across different sarcopenia statuses did not show evidence of significant nonlinearity (*p*‐nonlinearity > 0.05). As shown in Figure 2A–D, participants with impaired PEF consistently exhibited higher cumulative hazards of death compared with those with normal PEF across the overall population and within all sarcopenia subgroups (all log‐rank *p* < 0.001), with the separation of survival curves most pronounced among those with diagnosed sarcopenia. In the multivariable‐adjusted Cox models (Figure 2E; Table S1), impaired PEF (vs. normal PEF) was significantly associated with increased all‐cause mortality in the overall population (HR: 1.61, 95% CI: 1.43–1.81). After further adjustment for sarcopenia status, the association was slightly attenuated but remained significant (HR: 1.52, 95% CI: 1.35–1.72). No significant interaction was observed across sarcopenia subgroups (*p*‐interaction = 0.382). When modelled continuously, each SD decrease in PEF was associated with an increased mortality risk, with the strongest association observed in the diagnosed sarcopenia group (HR: 1.49, 95% CI: 1.30–1.71), followed by the nonsarcopenia (HR: 1.29, 95% CI: 1.18–1.41) and possible sarcopenia (HR: 1.15, 95% CI: 1.03–1.29) groups. However, the interaction test was not statistically significant (*p*‐interaction = 0.127).

**TABLE 2:** Association between sarcopenia and all‐cause death.

	Association of sarcopenia status with all‐cause death			*p*‐trend
	Nonsarcopenia	Possible sarcopenia	Diagnosed sarcopenia	
Events/total	626/7667	465/3002	445/1358	
Incidence rate	9.29 (8.59–10.05)	18.06 (16.50–19.78)	40.99 (37.36–44.98)	
Crude model	Ref.	1.94 (1.72–2.19)	4.63 (2.10–5.23)	< 0.001
Model 1	Ref.	1.44 (1.28–1.64)	1.93 (1.68–2.22)	< 0.001
Model 2	Ref.	1.51 (1.33–1.71)	1.67 (1.43–1.94)	< 0.001

*Note:* Model 1: adjusted for sex (male or female), age (continuous), residence (urban or rural), education level (below lower secondary, upper secondary and vocational training, or tertiary education), smoking habits (never, ever or current), alcohol consumption (yes or no), physical activity (active or inactive) and disease status (diabetes, hypertension, cardiovascular disease, arthritis, dyslipidemia and cancer; yes or no).

Model 2: additionally adjusted for body mass index (continuous).

The incidence rate indicates the number of deaths per 1000 person‐years.

**FIGURE 3:**
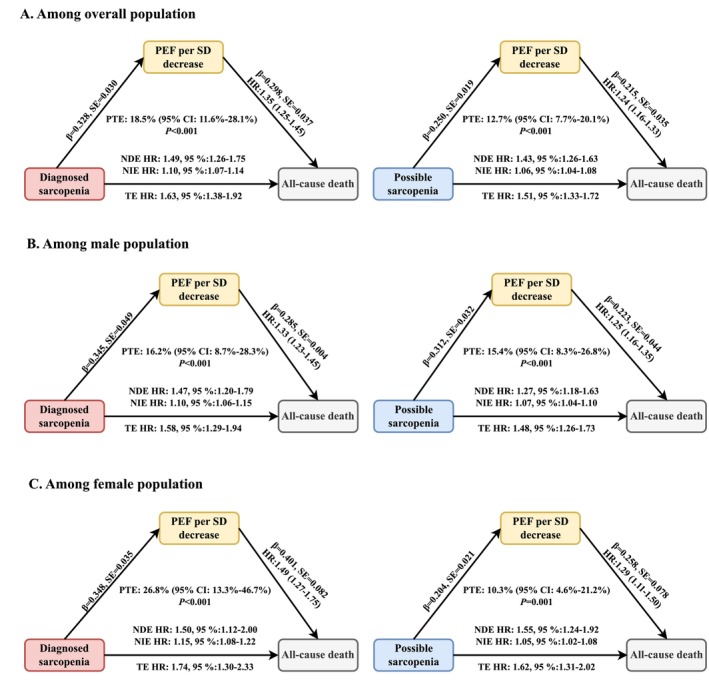
Causal mediation effects of sarcopenia status in the association between reduced peak expiratory flow (PEF) and all‐cause mortality in the overall population and by sex subgroups Panel **A**: Overall population; Panel **B**: Male population; Panel **C**: Female population. The mediation analyses were adjusted for sex (male or female; only in analysis of overall population), age (continuous), body mass index (continuous), residence (urban or rural), education level (below lower secondary, upper secondary and vocational training, or tertiary education), smoking habits (never, ever, or current), alcohol consumption (yes or no), physical activity (active or inactive) and disease status (diabetes, hypertension, cardiovascular disease, arthritis, dyslipidemia and cancer). NDE, natural direct effect; NIE, natural indirect effect; PEF, peak expiratory flow; PTE, proportion mediated; TE, total effect.

**FIGURE 4:**
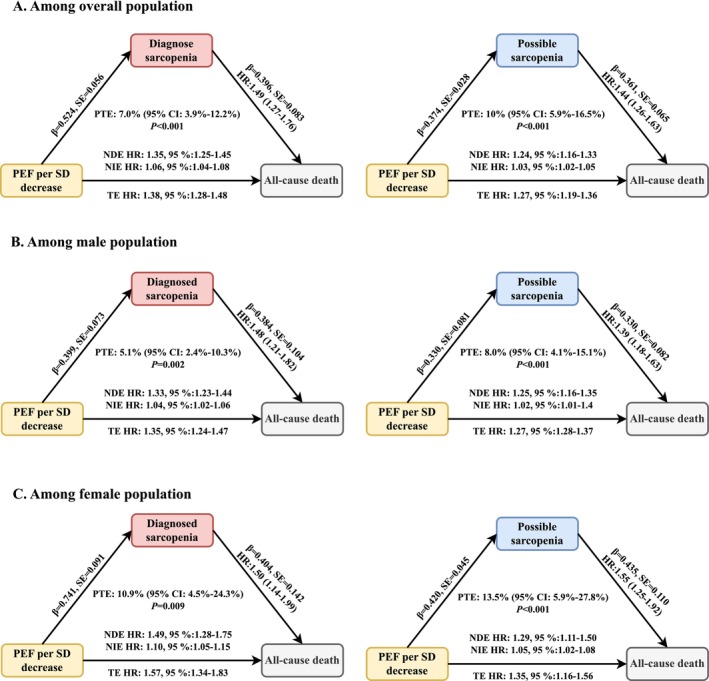
Causal mediation effects of reduced peak expiratory flow (PEF) in the association between sarcopenia status and all‐cause mortality in the overall population and by sex subgroups **Caption**: Panel **A**: Overall population; Panel **B**: Male population; Panel **C**: Female population. The mediation analyses were adjusted for sex (male or female; only in analysis of overall population), age (continuous), body mass index (continuous), residence (urban or rural), education level (below lower secondary, upper secondary and vocational training, or tertiary education), smoking habits (never, ever, or current), alcohol consumption (yes or no), physical activity (active or inactive), and disease status (diabetes, hypertension, cardiovascular disease, arthritis, dyslipidemia and cancer). NDE, natural direct effect; NIE, natural indirect effect; PEF, peak expiratory flow; PTE, proportion mediated; TE, total effect.

In the causal mediation analysis, both diagnosed and possible sarcopenia were associated with higher mortality, partly mediated by reduced PEF (Figure 3). In the overall population, diagnosed sarcopenia showed a TE of HR: 1.63 (95% CI: 1.38–1.92), which comprised an NDE of HR: 1.49 (95% CI: 1.26–1.75) and an NIE of HR: 1.10 (95% CI: 1.05–1.16), yielding a PTE of 18.5%. For possible sarcopenia, the corresponding estimates were TE: HR: 1.51 (95% CI: 1.33–1.72), NDE: HR: 1.43 (95% CI: 1.26–1.63), and NIE: HR: 1.06 (95% CI: 1.04–1.08), with a PTE of 12.7%. Conversely, when reduced PEF (per SD decrease) was considered the exposure (Figure 4), sarcopenia mediated a modest but significant proportion of its mortality risk. The TE was HR: 1.38 (95% CI: 1.28–1.48) with diagnosed sarcopenia as the mediator (NDE: HR: 1.35, 95% CI: 1.25–1.45; NIE: HR: 1.03, 95% CI: 1.02–1.05; PTE: 7.0%), and HR: 1.27 (95% CI: 1.19–1.36) with possible sarcopenia as the mediator (NDE: HR: 1.24, 95% CI: 1.16–1.33; NIE: HR: 1.03, 95% CI: 1.02–1.05; PTE: 10.0%). Sex‐specific analyses revealed stronger mediation through PEF in women with diagnosed sarcopenia (TE: HR 1.74, 95% CI: 1.30–2.33; NDE: HR: 1.50, 95% CI: 1.12–2.00; NIE: HR: 1.15, 95% CI: 1.08–1.22; PTE: 26.8%). By contrast, the effect of lower PEF on mortality was more strongly mediated by possible sarcopenia in both men (PTE: 8.0%) and women (PTE: 13.5%).

We constructed six subgroups to assess the joint effects of sarcopenia and impaired PEF on mortality (Table 3). Incidence rates rose stepwise from 6.00 per 1000 person‐years in the reference group (nonsarcopenia with normal PEF) to 46.72 per 1000 person‐years in those with both diagnosed sarcopenia and impaired PEF. After multivariable adjustment, mortality risks remained significantly elevated: HR: 1.52 (95% CI: 1.22–1.89) for possible sarcopenia with normal PEF, 1.73 (1.28–2.35) for diagnosed sarcopenia with normal PEF, 1.60 (1.36–1.88) for impaired PEF without sarcopenia, 2.22 (1.87–2.64) for impaired PEF with possible sarcopenia, and 2.44 (2.01–2.97) for impaired PEF with diagnosed sarcopenia.

**TABLE 3:** Association of all‐cause death with joint exposure to sarcopenia and impaired respiratory function in overall population and across sex subgroups.

	Joint associations of sarcopenia and impaired PEF with all‐cause death, HRs (95% CIs)					
	Normal PEF and nonsarcopenia	Normal PEF and possible sarcopenia	Normal PEF and diagnosed sarcopenia	Impaired PEF and nonsarcopenia	Impaired PEF and possible sarcopenia	Impaired PFE and diagnosed sarcopenia
**In overall population**						
Event/total	250/4696	123/1299	57/302	376/2971	342/1703	388/1056
Incidence rate	6.00 (5.30–6.79)	10.79 (9.04–12.88)	22.35 (17.24–28.98)	16.24 (14.53–18.16)	26.11 (23.28–29.29)	46.72 (42.29–51.61)
Crude model	Ref.	1.78 (1.43–2.21)	3.89 (2.92–5.19)	2.44 (2.08–2.86)	4.00 (3.39–4.70)	8.19 (6.98–9.60)
Model 1	Ref.	1.47 (1.19–1.83)	2.02 (1.50–2.71)	1.64 (1.39–1.93)	2.18 (1.83–2.59)	2.83 (2.36–3.39)
Model 2	Ref.	1.52 (1.22–1.89)	1.73 (1.28–2.35)	1.60 (1.36–1.88)	2.22 (1.87–2.64)	2.44 (2.01–2.97)
**In male population**						
Event/total	173/2389	71/514	34/131	273/1563	199/729	215/475
Incidence rate	8.22 (7.08–9.54)	15.86 (12.57–20.01)	31.58 (22.56–44.19)	20.50 (18.20–23.08)	33.09 (28.80–38.03)	60.37 (52.82–69.01)
Crude model	Ref.	1.90 (1.44–2.51)	4.15 (2.87–5.99)	2.52 (2.08–3.05)	4.10 (3.35–5.02)	7.85 (6.43–9.60)
Model 1	Ref.	1.53 (1.16–2.02)	2.26 (1.56–3.30)	1.76 (1.45–2.14)	2.23 (1.79–2.76)	3.06 (2.44–3.85)
Model 2	Ref.	1.56 (1.18–2.06)	1.98 (1.34–2.91)	1.72 (1.41–2.09)	2.25 (1.81–2.79)	2.68 (2.09–3.43)
**In female population**						
Event/total	77/2307	52/785	23/171	103/1408	143/974	173/581
Incidence rate	3.74 (2.99–4.67)	7.52 (5.73–9.86)	15.61 (10.37–23.49)	8.30 (6.84–10.07)	17.17 (14.57–20.22)	36.47 (31.42–42.33)
Crude model	Ref.	1.99 (1.40–2.83)	4.28 (2.68–6.81)	2.21 (1.65–2.98)	4.58 (3.47–6.04)	10.13 (7.74–13.25)
Model 1	Ref.	1.45 (1.02–2.06)	1.76 (1.09–2.83)	1.46 (1.08–1.97)	2.16 (1.61–2.88)	2.58 (1.90–3.51)
Model 2	Ref.	1.52 (1.07–2.18)	1.48 (0.91–2.41)	1.43 (1.05–1.93)	2.23 (1.67–2.99)	2.21 (1.60–3.05)

*Note:* *p*‐interaction: Sarcopenia–PEF subgroups * Gender (male, female) = 0.237.

Model 1: adjusted for sex (male or female; in the overall population analysis only), age (continuous), residence (urban or rural), education level (below lower secondary, upper secondary and vocational training, or tertiary education), smoking habits (never, ever, or current), alcohol consumption (yes or no), physical activity (active or inactive), and disease status (diabetes, hypertension, cardiovascular disease, arthritis, dyslipidemia and cancer).

Model 2: additionally adjusted for body mass index (continuous).

The incidence rate indicates the number of deaths per 1000 person‐years.

Abbreviations: CI, confidence interval; HR, hazard ratio; PEF, peak expiratory flow.

Sex‐stratified analyses revealed broadly consistent patterns, with no significant multiplicative interaction (Table 3; *p*‐interaction = 0.237). Among men, the highest mortality risk was observed in those with both impaired PEF and diagnosed sarcopenia (HR: 2.68, 95% CI: 2.09–3.43), while the corresponding risk estimate among women was 2.21 (95% CI: 1.60–3.05). Age‐stratified analyses showed a stronger joint effect in participants < 60 years (HR: 5.32, 95% CI: 3.37–8.40) compared with those ≥ 60 years (HR: 3.57, 95% CI: 2.87–4.44), although no significant interaction was detected (*p*‐interaction = 0.255; Table S2).

Across lifestyle subgroups, mortality risk increased progressively with greater severity of sarcopenia and impaired PEF, with no significant heterogeneity by smoking, drinking, physical activity, or ideal lifestyle status (all *p*‐interaction > 0.05; Table 4; Tables S3–S6). In smoking‐stratified analyses, the joint exposure of diagnosed sarcopenia and impaired PEF conferred similarly elevated risks among never‐smokers (HR: 2.27), ever‐smokers (HR: 2.23), and current smokers (HR: 2.71). Although participants with an ideal lifestyle pattern had lower absolute mortality rates, the relative risks associated with the joint exposure were comparable to those without an ideal lifestyle. Sensitivity analyses yielded consistent findings after excluding deaths within the first year of follow‐up and participants with respiratory disease or arthritis (Table S7). Additionally, the findings remained consistent when impaired PEF was redefined using the Chinese criteria (Table S8).

**TABLE 4:** Stratified analyses of the association between joint exposure to sarcopenia and impaired respiratory function with death by lifestyle patterns.

	Incidence rates	Joint associations of sarcopenia and impaired PEF with all‐cause death, HRs (95% CIs)					
		Normal PEF and nonsarcopenia	Normal PEF and possible sarcopenia	Normal PEF and diagnosed sarcopenia	Impaired PEF and nonsarcopenia	Impaired PEF and possible sarcopenia	Impaired PEF and diagnosed sarcopenia
**Stratified by smoking status** ^**a**^ **:** *P*‐INTm = 0.272							
Never smokers	11.07 (10.28–11.92)	Ref.	1.45 (1.06–1.99)	1.44 (0.91–2.28)	1.39 (1.07–1.80)	2.24 (1.74–2.87)	2.27 (1.69–3.04)
Former smokers	26.32 (23.13–29.94)	Ref.	1.03 (0.53–1.98)	2.69 (1.28–5.63)	1.56 (1.03–2.37)	1.92 (1.23–3.00)	2.23 (1.31–3.79)
Current smokers	18.86 (17.42–20.42)	Ref.	1.84 (1.31–2.60)	1.85 (1.13–3.03)	1.85 (1.44–2.37)	2.31 (1.74–3.07)	2.71 (1.99–3.68)
**Stratified by drinking status** ^**b**^ **:** *P*‐INTm = 0.258							
Yes	15.75 (14.49–17.13)	Ref.	1.71 (1.21–2.43)	1.57 (0.92–2.70)	1.78 (1.39–2.29)	2.18 (1.63–2.90)	2.41 (1.74–3.34)
No	14.28 (13.41–15.20)	Ref.	1.44 (1.09–1.90)	1.84 (1.27–2.67)	1.48 (1.19–1.85)	2.22 (1.79–2.76)	2.47 (1.94–3.16)
**Stratified by physical activity** ^**c**^ **:** *P*‐INTm = 0.726							
Active	10.12 (8.96–11.44)	Ref.	1.71 (1.05–2.79)	0.68 (0.27–1.76)	1.30 (0.91–1.86)	1.93 (1.30–2.87)	2.21 (1.40–3.49)
Inactive	16.28 (15.41–17.20)	Ref.	1.49 (1.17–1.91)	2.05 (1.48–2.84)	1.68 (1.39–2.02)	2.29 (1.89–2.77)	2.51 (2.02–3.13)
**Stratified by ideal lifestyle pattern** ^**d**^ **:** *P*‐INTm = 0.644							
Yes	6.63 (5.38–8.18)	Ref.	1.80 (0.80–4.03)	—	1.01 (0.50–1.99)	2.37 (1.26–4.49)	1.99 (0.88–4.48)
No	15.95 (15.14–16.79)	Ref.	1.52 (1.21–1.90)	1.86 (1.37–2.53)	1.67 (1.41–1.98)	2.23 (1.86–2.67)	2.48 (2.03–3.03)

*Note:* All models were adjusted for sex (male or female), age (continuous), body mass index (continuous), residence (urban or rural), education level (below lower secondary, upper secondary and vocational training, or tertiary education), smoking habit (never, ever or current), alcohol consumption (yes or no), physical activity (active or inactive) and disease status (diabetes, hypertension, cardiovascular disease, arthritis, dyslipidemia and cancer).

Ideal lifestyle pattern was defined as no smoking, no drinking and being physically active.

Abbreviations: CI, confidence interval; HR, hazard ratio; INTm, multiplicative interaction; PEF, peak expiratory flow.

^a^ Excluding smoking habit in the multivariable model.

^b^ Excluding alcohol consumption in the multivariable model.

^c^ Excluding physical activity in the multivariable model.

^d^ Excluding smoking habit, alcohol consumption and physical activity in the multivariable model.

## 4 Discussion

Based on data from a national cohort in China, this study is the first to demonstrate a causal bidirectional mediation between sarcopenia and impaired PEF on the risk for all‐cause mortality, with distinct patterns for diagnosed and possible sarcopenia. Each SD decrease in PEF was associated with 29%, 15% and 49% higher mortality risks in nonsarcopenia, possible sarcopenia and diagnosed sarcopenia groups, respectively, although the interaction was not statistically significant. In the causal mediation analysis, diagnosed sarcopenia exerted its effect on mortality largely through reduced PEF, accounting for 18.5% of the excess risk, compared with 12.7% for possible sarcopenia. Conversely, when PEF was treated as the exposure, its adverse effect on mortality was more strongly mediated by possible sarcopenia (10.0%) than by diagnosed sarcopenia (7.0%). Furthermore, joint exposure to sarcopenia and impaired PEF substantially increased mortality risk, with no significant heterogeneity across sex, age or lifestyle subgroups.

Compelling evidence from previous studies has consistently revealed a significant association between sarcopenia [11, 12, 13, 14], diminished respiratory function [15, 30] and all‐cause death, which is in line with our findings. A previous study that used the same dataset demonstrated a similar mortality risk associated with sarcopenia during a 7‐year follow‐up as our study [11]. Sarcopenia is characterized by a progressive loss of muscle mass and function and is closely related to compromised immune function [6], staggering, falls and even frailty in elderly individuals [7, 8], increasing their susceptibility to various life‐threatening complications [6, 31]. Impaired respiratory function, indicated by a reduced PEF, reflects diminished lung capacity and respiratory muscle strength, which can lead to insufficient oxygenation of vital organs and increased vulnerability to respiratory infections and cardiovascular events [25, 32, 33]. Therefore, sarcopenia and impaired respiratory function appear to act synergistically, contributing to increased mortality risk through their combined adverse impact on overall physical health and resilience.

Importantly, our findings highlight distinct roles of diagnosed and possible sarcopenia in the PEF–mortality pathway. When impaired PEF was treated as a categorical variable, mortality risks appeared broadly similar across sarcopenia strata, with slightly attenuated HRs at greater sarcopenia severity. This likely reflects the limited sensitivity of fixed cutoffs in populations where mean PEF values are already low. In contrast, modelling PEF continuously (per‐SD decrease) revealed substantially stronger excess risks among individuals with diagnosed sarcopenia but weaker associations in those with possible sarcopenia. Our causal mediation analysis provides mechanistic support for these patterns: diagnosed sarcopenia exerted its effect on mortality primarily through reduced PEF, whereas the adverse effect of reduced PEF on mortality was more strongly mediated through possible sarcopenia. These results reinforce the prognostic value of functional phenotypes, consistent with emerging evidence linking muscle strength and physical performance to adverse health outcomes [34, 35]. For example, the Otassha Study in Japan reported that respiratory sarcopenia independently predicted all‐cause mortality among community‐dwelling older adults, even in the absence of confirmed muscle mass loss [35]. Together, the results emphasize the intertwined roles of sarcopenia status and respiratory health and highlight the importance of assessing both to identify individuals at highest risk.

Several mechanisms may underlie the bidirectional mediation effect of impaired respiratory function and sarcopenia on mortality. Both conditions are closely linked to aging‐related pathologies, chronic systemic inflammation, excessive oxidative stress and mitochondrial dysfunction, all of which exacerbate declines in muscle and lung function [36, 37]. Importantly, sarcopenia and impaired respiratory function likely interact, compounding mortality risk. The progressive loss of skeletal muscle strength extends to respiratory muscles, including the rectus abdominis and the internal and external obliques, leading to their atrophy and consequent reductions in lung capacity and respiratory efficiency [38]. This systemic impact may partly explain why sarcopenia contributes to health deterioration and increased all‐cause mortality. Supporting this, sarcopenia has been associated with higher risks of pneumonia [10], lung function decline [19, 20] and mortality in aspiration pneumonia [14]. Conversely, impaired PEF serves as a sentinel marker of lung health and overall physiological resilience during aging [30] and also captures both reduced respiratory muscle performance and the indirect effects of chronic conditions such as COPD [39], which themselves are accompanied by musculoskeletal complications including sarcopenia and cachexia. Together, these reciprocal influences provide mechanistic plausibility for the observed synergistic impact of sarcopenia and impaired respiratory function on mortality. Further mechanistic studies are warranted to delineate these pathways.

### 4.1 Implications for Clinical Practice and the Healthcare System

Given the strong associations between sarcopenia, impaired PEF, and increased mortality, routine screening for both conditions in adults over 45 years should be integrated into standard healthcare practice. In line with the emerging concept of ‘respiratory sarcopenia,’ simple measures of respiratory function (such as PEF) together with functional performance tests may provide a practical approach for early risk stratification, particularly in settings where full diagnostic assessments are not available [35, 40]. Early identification enables timely interventions that could reduce mortality risk. Our causal mediation analyses further demonstrated distinct roles of different sarcopenia stages in the PEF–mortality pathway, underscoring the importance of combining sarcopenia staging with respiratory function assessment to more accurately identify high‐risk individuals. Although formal interaction tests did not reveal statistically significant effect modification by lifestyle factors, participants with smoking and physical inactivity had higher baseline mortality rates. This suggests that while lifestyle factors may not directly alter the joint impact of sarcopenia and impaired PEF, they remain important population‐level targets for prevention and health promotion. For individuals with coexisting sarcopenia and ventilatory decline, clinical care should emphasize integrated management of muscle and respiratory health. Healthcare providers may consider comprehensive care plans incorporating physical rehabilitation, respiratory therapy and nutritional support [1], with special attention to individuals identified at heightened risk through combined sarcopenia–PEF screening.

This study has several key strengths. First, it draws on data from a large, nationally representative cohort, enhancing the validity and generalizability of the findings within the Chinese population. Second, this is the first study to comprehensively investigate the joint effects of sarcopenia and impaired respiratory function on mortality via rigorous statistical methods, including interaction analysis, causal mediation analysis, extensive subgroup analyses and sensitivity tests. These methodological approaches provide deeper insight into the mechanisms linking these conditions and help identify key factors contributing to excess mortality risk. Other merits include the substantial sample size, long follow‐up period, high‐quality data and prospective cohort design.

However, there are several limitations to consider. First, the study population consisted of middle‐aged and older adults from China, which may limit the generalizability of the findings to other populations with different demographic or ethnic characteristics. Second, ASM was estimated using anthropometric equations incorporating height and weight rather than direct measurements such as DXA. While practical for large epidemiological studies [6, 22], this approach is less precise and may be influenced by BMI, potentially introducing measurement bias. Third, covariates were collected via self‐reported standardized questionnaires, which may have led to recall bias and affected the accuracy and interpretability of the findings. Fourth, sarcopenia status and PEF were assessed at baseline only, precluding evaluation of their longitudinal changes over time. Future studies incorporating repeated assessments, time‐varying analytical frameworks, and more objective measurements across diverse populations are warranted to strengthen the clinical applicability of these findings.

## 5 Conclusions

This study identified a significant bidirectional mediation effect of sarcopenia and impaired PEF on death among middle‐aged and elderly Chinese adults, underscoring their interconnection and joint contribution to excess mortality risk. The findings highlight the importance of combined assessment and integrated management of muscle and respiratory health.

## Funding

This study was supported by the Natural Science Foundation of China (No. 82271853) and the Zhongnanshan Medical Foundation of Guangdong (No. ZNSXS‐20240011), the Special Fund Project for Science and Technology Innovation Strategy of Guangdong Province (No. 202053–75 and 202053–74), and the Shantou Science and Technology Plan Project, Medical and Health Category (No. 250720056494466 and 250729116497223). The study funders were not involved in the design of the study; the collection, analysis, and interpretation of the data; or the publication of the report.

## Ethics Statement

The CHARLS study was approved by the Biomedical Ethics Review Committee of Peking University (IRB00001052–11015) and has therefore been performed in accordance with ethical standards laid down in the 1964 Declaration of Helsinki and its later amendments.

## Consent

All participants provided written informed consent prior to their enrolment.

## Conflicts of Interest

The authors declare no conflicts of interest.

## Supporting information


**Table S1:** Association of all‐cause death with respiratory function defined by CAPTURE Criteria.
**Table S2:** Association of all‐cause death with joint exposure to sarcopenia and CAPTURE‐defined impaired respiratory function stratified by age.
**Table S3:** Association of all‐cause death with joint exposure to sarcopenia and CAPTURE‐defined impaired respiratory function stratified by smoking habits.
**Table S4:** Association of all‐cause death with joint exposure to sarcopenia and CAPTURE‐defined impaired respiratory function stratified by physical activity.
**Table S5:** Association of all‐cause death with joint exposure to sarcopenia and CAPTURE‐defined impaired respiratory function stratified by drinking habits.
**Table S6:** Association of all‐cause death with joint exposure to sarcopenia and CAPTURE‐defined impaired respiratory function stratified by ideal lifestyle pattern.
**Table S7:** Sensitivity analysis.
**Table S8:** Association of all‐cause death with joint exposure to sarcopenia and Chinese criteria‐defined impaired respiratory function.
**Figure S1:** Flowchart of the study population.
**Figure S2:** Multivariable dose–response associations between peak expiratory flow (PEF) and all‐cause mortality across sarcopenia subgroups stratified by and sex.

## Data Availability

The datasets used and/or analysed during the current study are publicly available or from the corresponding author upon reasonable request.
