# Supplementary material for: Combined Effect of Sarcopenia and Impaired Respiratory Function on All‐Cause Death: A Nationwide Cohort Study
Source: J Cachexia Sarcopenia Muscle. 2026 Apr 5;17(2):e70275. doi: 10.1002/jcsm.70275 (PMC13051881; doi:10.1002/jcsm.70275)
Supplement: Supplementary file 1 — Table S1: Association of all‐cause death with respiratory function defined by CAPTURE Criteria. Table S2: Association of all‐cause death with joint exposure to sarcopenia and CAPTURE‐defined impaired respiratory function stratified by age. Table S3: Association of all‐cause death with joint exposure to sarcopenia and CAPTURE‐defined impaired respiratory function stratified by smoking habits. Table S4: Association of all‐cause death with joint exposure to sarcopenia and CAPTURE‐defined impaired respiratory function stratified by physical activity. Table S5: Association of all‐cause death with joint exposure to sarcopenia and CAPTURE‐defined impaired respiratory function stratified by drinking habits. Table S6: Association of all‐cause death with joint exposure to sarcopenia and CAPTURE‐defined impaired respiratory function stratified by ideal lifestyle pattern. Table S7: Sensitivity analysis. Table S8: Association of all‐cause death with joint exposure to sarcopenia and Chinese criteria‐defined impaired respiratory function. Figure S1: Flowchart of the study population. Figure S2: Multivariable dose–response associations between peak expiratory flow (PEF) and all‐cause mortality across sarcopenia subgroups stratified by and sex. [file JCSM-17-e70275-s001.docx]

**Supplementary Materials**

[**Supplementary eTable 1.** Association of all-cause death with respiratory function defined by CAPTURE Criteria 2](#_Toc219833889)

[**Supplementary eTable 2.** Association of all-cause death with joint exposure to sarcopenia and CAPTURE-defined impaired respiratory function stratified by age 3](#_Toc219833890)

[**Supplementary eTable 3.** Association of all-cause death with joint exposure to sarcopenia and CAPTURE-defined impaired respiratory function stratified by smoking habits 4](#_Toc219833891)

[**Supplementary eTable 4.** Association of all-cause death with joint exposure to sarcopenia and CAPTURE-defined impaired respiratory function stratified by physical activity 6](#_Toc219833892)

[**Supplementary eTable 5.** Association of all-cause death with joint exposure to sarcopenia and CAPTURE-defined impaired respiratory function stratified by drinking habits 7](#_Toc219833893)

[**Supplementary eTable 6.** Association of all-cause death with joint exposure to sarcopenia and CAPTURE-defined impaired respiratory function stratified by ideal lifestyle pattern 8](#_Toc219833894)

[**Supplementary eTable 7.** Sensitivity analysis 9](#_Toc219833895)

[**Supplementary eTable 8.** Association of all-cause death with joint exposure to sarcopenia and Chinese criteria-defined impaired respiratory function 11](#_Toc219833896)

[**Supplementary eFigure 1.** Flowchart of the study population 12](#_Toc219833897)

[**Supplementary eFigure 2.** Multivariable dose–response associations between peak expiratory flow (PEF) and all-cause mortality across sarcopenia subgroups stratified by and sex 14](#_Toc219833898)

[**Supplementary eMethods** 15](#_Toc219833899)

# **Supplementary eTable 1.** Association of all-cause death with respiratory function defined by CAPTURE Criteria

|  | **Association of PEF-indicated respiratory function**  **with all-cause death, HRs (95% CIs)** | | ***P*-trend** | **Per SD decrease in PEF** |
| --- | --- | --- | --- | --- |
|  | **Normal PEF** | **Impaired PEF** |  |  |
| Event/Total | 430/6297 | 1106/5790 |  |  |
| Incidence rate | 7.7 (7.0‒8.5) | 22.9 (21.6‒24.2) |  |  |
| Crude model | Reference | 2.99 (2.68‒3.35) | <0.001 | 1.67 (1.58‒1.76) |
| Model 1 | Reference | 1.66 (1.47‒1.87) | <0.001 | 1.35 (1.27‒1.44) |
| Model 2 | Reference | 1.61 (1.43‒1.81) | <0.001 | 1.33 (1.25‒1.41) |
| Model 3 | Reference | 1.60 (1.42‒1.80) | <0.001 | 1.33 (1.25‒1.41) |
| **Model 4** | **Reference** | **1.52 (1.35‒1.72)** | **<0.001** | **1.30 (1.22‒1.38)** |

Model 1: adjusted for sex (male or female), age (continuous), residence (urban or rural), education level (below lower secondary, upper secondary and vocational training, or tertiary education), smoking habits (never, ever or current), alcohol consumption (yes or no), physical activity (active or inactive), and disease status (diabetes, hypertension, cardiovascular disease, arthritis, dyslipidemia, cancer).

Model 2: additionally adjusted for body mass index (continuous) on the basis of Model 1.

Model 3: excluding sex in Model 2.

**Model 4: additionally adjusted for sarcopenia status (non-sarcopenia, possible or diagnosed sarcopenia) on the basis of Model 2.**

Per SD: mortality risk per-SD increase in PEF (125.43 L/min).

Abbreviations: CAPTURE, chronic obstructive pulmonary disease assessment in primary care to identify undiagnosed respiratory disease and exacerbation risk; PEF, peak expiratory flow; SD, standard deviation.

# **Supplementary eTable 2.** Association of all-cause death with joint exposure to sarcopenia and CAPTURE-defined impaired respiratory function stratified by age

|  | **Joint associations of sarcopenia and impaired PEF with all-cause death, HRs (95% CIs)** | | | | | | ***P*-INTm** |
| --- | --- | --- | --- | --- | --- | --- | --- |
|  | **Normal PEF & Non-sarcopenia** | **Normal PEF & Possible sarcopenia** | **Normal PEF & Diagnosed sarcopenia** | **Impaired PEF & Non-sarcopenia** | **Impaired PEF & Possible sarcopenia** | **Impaired PFE & Diagnosed sarcopenia** |  |
| **<60 years** (360/6810) | | | | | | | **0.255** |
| Event/Total | 112/3471 | 47/819 | 7/97 | 105/1581 | 61/704 | 28/138 |  |
| Incidence rate | 3.61 (3.00–4.35) | 6.46 (4.85–8.60) | 8.35 (3.98–17.52) | 7.52 (6.21–9.11) | 9.15 (7.71–12.74) | 24.20 (16.71–35.05) |  |
| Crude model | Reference | 1.76 (1.25–2.47) | 2.40 (1.12–5.15) | 2.08 (1.60–2.72) | 2.71 (1.99–3.71) | 6.72 (4.44–10.17) |  |
| Model 1 | Reference | 1.83 (1.30–2.58) | 2.75 (1.28–5.92) | 2.03 (1.55–2.65) | 2.72 (1.98–3.73) | 7.20 (4.73–10.96) |  |
| Model 2 | Reference | 1.87 (1.33‒2.64) | 2.10 (0.96‒4.59) | 1.94 (1.48‒2.53) | 2.68 (1.96‒3.68) | 5.32 (3.37‒8.40) |  |
| **≥60 years** (1176/5217) | | | | | | |  |
| Event/Total | 138/1225 | 76/480 | 50/205 | 271/1390 | 281/999 | 360/918 |  |
| Incidence rate | 12.96 (10.97–15.31) | 18.44 (14.73–23.09) | 29.20 (22.13–38.53) | 23.02 (20.44–25.93) | 34.33 (30.52–38.56) | 50.36 (45.42–55.85) |  |
| Crude model | Reference | 1.42 (1.07–1.87) | 2.34 (1.69–3.23) | 1.77 (1.44–2.18) | 2.67 (2.18–3.28) | 4.08 (3.35–4.96) |  |
| Model 1 | Reference | 1.40 (1.06–1.86) | 2.50 (1.80–3.46) | 1.68 (1.37–2.06) | 2.50 (2.04–3.09) | 4.10 (3.36–5.01) |  |
| Model 2 | Reference | 1.45 (1.09‒1.92) | 2.16 (1.54‒3.03) | 1.65 (1.34‒2.02) | 2.55 (2.08‒3.14) | 3.57 (2.87‒4.44) |  |

Model 1: adjusted for sex (male or female), age (continuous), residence (urban or rural), education level (below lower secondary, upper secondary and vocational training, or tertiary education), smoking habits (never, ever or current), alcohol consumption (yes or no), physical activity (active or inactive) and disease status (diabetes, hypertension, cardiovascular disease, arthritis, and dyslipidemia and cancer).

Model 2: additionally adjusted for body mass index (continuous).

The incidence rate indicates the number of deaths per 1000 person-years.

Abbreviations: CAPTURE, chronic obstructive pulmonary disease assessment in primary care to identify undiagnosed respiratory disease and exacerbation risk; CI, confidence interval; HR, hazard ratio; INTm, multiplicative interaction; PEF, peak expiratory flow.

# **Supplementary eTable 3.** Association of all-cause death with joint exposure to sarcopenia and CAPTURE-defined impaired respiratory function stratified by smoking habits

|  | **Joint associations of sarcopenia and impaired PEF with all-cause death, HRs (95% CIs)** | | | | | | ***P*-INTm** |
| --- | --- | --- | --- | --- | --- | --- | --- |
|  | **Normal PEF & Non-sarcopenia** | **Normal PEF & Possible sarcopenia** | **Normal PEF &** **Diagnosed sarcopenia** | **Impaired PEF & Non-sarcopenia** | **Impaired PEF & Possible sarcopenia** | **Impaired PFE & Diagnosed sarcopenia** |  |
| **Never smokers** (697/7202) | | | | | | | **0.272** |
| Event/Total | 110/2828 | 61/850 | 25/183 | 129/1633 | 178/1086 | 199/625 |  |
| Incidence rate | 4.36 (3.62–5.26) | 8.14 (6.33–10.46) | 15.85 (10.71–23.46) | 8.98 (7.55–10.67) | 19.19 (16.56–22.22) | 39.55 (34.42–45.45) |  |
| Crude model | Reference | 1.84 (1.35–2.52) | 3.76 (2.44–5.81) | 2.07 (1.60–2.67) | 4.41 (3.48–5.60) | 9.49 (7.52–11.99) |  |
| Model 1 | Reference | 1.40 (1.02–1.92) | 1.66 (1.07–2.60) | 1.37 (1.06–1.78) | 2.14 (1.66–2.75) | 2.36 (1.76–3.16) |  |
| Model 2 | Reference | 1.45 (1.06–1.99) | 1.44 (0.91–2.28) | 1.39 (1.07–1.80) | 2.24 (1.74–2.87) | 2.27 (1.69–3.04) |  |
| **Ever smokers** (231/1060) | | | | | | |  |
| Event/Total | 39/378 | 12/108 | 11/21 | 62/296 | 59/172 | 45/88 |  |
| Incidence rate | 11.92 (8.71–16.31) | 12.87 (7.31–22.66) | 66.55 (36.86–120.17) | 25.27 (19.70–32.42) | 43.80 (33.93–66.53) | 70.46 (52.61–94.37) |  |
| Crude model | Reference | 1.09 (0.57–2.08) | 6.07 (3.11–11.86) | 2.10 (1.41–3.15) | 3.69 (2.46–5.53) | 6.15 (4.01–9.45) |  |
| Model 1 | Reference | 1.02 (0.53–1.96) | 2.88 (1.41–5.89) | 1.58 (1.04–2.39) | 1.91 (1.22–2.98) | 2.40 (1.46–3.93) |  |
| Model 2 | Reference | 1.03 (0.53–1.98) | 2.69 (1.28–5.63) | 1.56 (1.03–2.37) | 1.92 (1.23–3.00) | 2.23 (1.31–3.79) |  |
| **Current smokers** (608/3765) | | | | | | |  |
| Event/Total | 101/1490 | 50/341 | 21/98 | 185/1042 | 105/445 | 144/343 |  |
| Incidence rate | 7.67 (6.32–9.33) | 16.84 (12.76–22.22) | 25.99 (16.95–39.86) | 20.77 (17.98–23.99) | 28.23 (23.32–34.18) | 54.64 (46.41–64.34) |  |
| Crude model | Reference | 2.17 (1.55–3.05) | 3.58 (2.23–5.72) | 2.71 (2.13–3.45) | 3.74 (2.85–4.91) | 7.57 (5.87–9.77) |  |
| Model 1 | Reference | 1.78 (1.26–2.50) | 2.23 (1.38–3.59) | 1.93 (1.51–2.47) | 2.27 (1.71–3.01) | 3.24 (2.44–4.31) |  |
| Model 2 | Reference | 1.84 (1.31–2.60) | 1.85 (1.13–3.03) | 1.85 (1.44–2.37) | 2.31 (1.74–3.07) | 2.71 (1.99–3.68) |  |

Model 1: adjusted for sex (male or female), age (continuous), residence (urban or rural), education level (below lower secondary, upper secondary and vocational training, or tertiary education), alcohol consumption (yes or no), physical activity (active or inactive) and disease status (diabetes, hypertension, cardiovascular disease, arthritis, dyslipidemia and cancer).

Model 2: additionally adjusted for body mass index (continuous).

Abbreviations: CAPTURE, chronic obstructive pulmonary disease assessment in primary care to identify undiagnosed respiratory disease and exacerbation risk; CI, confidence interval; HR, hazard ratio; INTm, multiplicative interaction; PEF, peak expiratory flow.

# **Supplementary eTable 4.** Association of all-cause death with joint exposure to sarcopenia and CAPTURE-defined impaired respiratory function stratified by physical activity

|  | **Joint associations of sarcopenia and impaired PEF with all-cause death, HRs (95% CIs)** | | | | | | ***P*-INTm** |
| --- | --- | --- | --- | --- | --- | --- | --- |
|  | **Normal PEF & Non-sarcopenia** | **Normal PEF & Possible sarcopenia** | **Normal PEF & Diagnosed sarcopenia** | **Impaired PEF & Non-sarcopenia** | **Impaired PEF & Possible sarcopenia** | **Impaired PFE & Diagnosed sarcopenia** |  |
| **Physically active** (258/2899) | | | | | | | 0.726 |
| Event/Total | 58/1206 | 23/292 | 5/82 | 67/765 | 51/379 | 54/185 |  |
| Incidence rate | 5.41 (4.18–6.99) | 8.92 (5.93–13.42) | 6.92 (2.88–16.63) | 9.99 (7.86–12.69) | 15.94 (12.11–20.97) | 34.88 (26.72–45.54) |  |
| Crude model | Reference | 1.62 (1.00–2.62) | 1.40 (0.56–3.49) | 1.85 (1.30–2.63) | 2.87 (1.97–4.19) | 6.67 (4.61–9.67) |  |
| Model 1 | Reference | 1.67 (1.02‒2.71) | 0.87 (0.34‒2.19) | 1.34 (0.94‒1.92) | 1.85 (1.25‒2.75) | 2.81 (1.86‒4.26) |  |
| Model 2 | Reference | 1.71 (1.05–2.79) | 0.68 (0.27–1.76) | 1.30 (0.91–1.86) | 1.93 (1.30–2.87) | 2.21 (1.40–3.49) |  |
| **Physically inactive** (1278/9128) | | | | | | |  |
| Event/Total | 192/3490 | 100/1007 | 52/220 | 309/2206 | 291/1334 | 334/871 |  |
| Incidence rate | 6.21 (5.39–7.15) | 11.34 (9.32–13.80) | 28.45 (21.68–37.34) | 16.24 (14.53–18.16) | 19.19 (16.56–22.22) | 49.43 (44.40–55.02) |  |
| Crude model | Reference | 1.81 (1.42–2.30) | 4.74 (3.49–6.44) | 2.62 (2.19–3.14) | 4.26 (3.55–5.11) | 8.40 (7.03–10.03) |  |
| Model 1 | Reference | 1.45 (1.14‒1.85) | 2.35 (1.72‒3.22) | 1.72 (1.43‒2.06) | 2.25 (1.86‒2.73) | 2.86 (2.33‒3.50) |  |
| Model 2 | Reference | 1.49 (1.17–1.91) | 2.05 (1.48–2.84) | 1.68 (1.39–2.02) | 2.29 (1.89–2.77) | 2.51 (2.02–3.13) |  |

Model 1: adjusted for sex (male or female), age (continuous), residence (urban or rural), education level (below lower secondary, upper secondary and vocational training, or tertiary education), alcohol consumption (yes or no), smoking habits (never, ever or current), and disease status (diabetes, hypertension, cardiovascular disease, arthritis, dyslipidemia and cancer).

Model 2: additionally adjusted for body mass index (continuous).

Abbreviations: CAPTURE, chronic obstructive pulmonary disease assessment in primary care to identify undiagnosed respiratory disease and exacerbation risk; CI, confidence interval; HR, hazard ratio; INTm, multiplicative interaction; PEF, peak expiratory flow

# **Supplementary eTable 5.** Association of all-cause death with joint exposure to sarcopenia and CAPTURE-defined impaired respiratory function stratified by drinking habits

|  | **Joint associations of sarcopenia and impaired PEF with all-cause death, HRs (95% CIs)** | | | | | | ***P*-INTm** |
| --- | --- | --- | --- | --- | --- | --- | --- |
|  | **Normal PEF & Non-sarcopenia** | **Normal PEF & Possible sarcopenia** | **Normal PEF & Diagnosed sarcopenia** | **Impaired PEF & Non-sarcopenia** | **Impaired PEF & Possible sarcopenia** | **Impaired PFE & Diagnosed sarcopenia** |  |
| **Current drinkers** (547/4019) | | | | | | | 0.258 |
| Event/Total | 107/1732 | 46/359 | 17/87 | 170/1103 | 99/441 | 109/297 |  |
| Incidence rate | 6.98 (5.78–8.44) | 14.65 (10.97–19.56) | 23.15 (14.39–37.25) | 17.92 (15.42–20.83) | 26.75 (21.96–32.58) | 46.84 (38.82–56.51) |  |
| Crude model | Reference | 2.08 (1.47–2.94) | 3.62 (2.17–6.04) | 2.59 (2.03–3.30) | 3.92 (2.98–5.16) | 7.16 (5.48–9.35) |  |
| Model 1 | Reference | 1.70 (1.20‒2.40) | 1.68 (1.00‒2.85) | 1.81 (1.41‒2.32) | 2.17 (1.62‒2.89) | 2.56 (1.89‒3.47) |  |
| Model 2 | Reference | 1.71 (1.21–2.43) | 1.57 (0.92–2.70) | 1.78 (1.39–2.29) | 2.18 (1.63–2.90) | 2.41 (1.74–3.34) |  |
| **Non-current drinkers** (989/8008) | | | | | | |  |
| Event/Total | 143/2964 | 77/940 | 40/215 | 206/1868 | 243/1262 | 279/759 |  |
| Incidence rate | 5.43 (4.61–6.40) | 9.33 (7.46–11.66) | 22.03 (16.16–30.02) | 12.69 (11.06–14.54) | 22.38 (20.13–25.89) | 46.67 (41.50–52.48) |  |
| Crude model | Reference | 1.69 (1.28–2.23) | 4.17 (2.94–5.93) | 2.33 (1.89–2.89) | 4.20 (3.41–5.16) | 8.97 (7.33–10.97) |  |
| Model 1 | Reference | 1.38 (1.04‒1.82) | 2.20 (1.54‒3.15) | 1.51 (1.22‒1.88) | 2.17 (1.74‒2.69) | 2.94 (2.33‒3.69) |  |
| Model 2 | Reference | 1.44 (1.09–1.90) | 1.84 (1.27–2.67) | 1.48 (1.19–1.85) | 2.22 (1.79–2.76) | 2.47 (1.94–3.16) |  |

Model 1: adjusted for sex (male or female), age (continuous), residence (urban or rural), education level (below lower secondary, upper secondary and vocational training, or tertiary education), smoking habits (never, ever or current), physical activity (active or inactive) and disease status (diabetes, hypertension, cardiovascular disease, arthritis, dyslipidemia and cancer).

Model 2: additionally adjusted for body mass index (continuous).

Abbreviations: CAPTURE, chronic obstructive pulmonary disease assessment in primary care to identify undiagnosed respiratory disease and exacerbation risk; CI, confidence interval; HR, hazard ratio; INTm, multiplicative interaction; PEF, peak expiratory flow.

# **Supplementary eTable 6.** Association of all-cause death with joint exposure to sarcopenia and CAPTURE-defined impaired respiratory function stratified by ideal lifestyle pattern

|  | **Joint associations of sarcopenia and impaired PEF with all-cause death, HRs (95% CIs)** | | | | | | ***P*-INTm** |
| --- | --- | --- | --- | --- | --- | --- | --- |
|  | **Normal PEF & Non-sarcopenia** | **Normal PEF & Possible sarcopenia** | **Normal PEF & Diagnosed sarcopenia** | **Impaired PEF & Non-sarcopenia** | **Impaired PEF & Possible sarcopenia** | **Impaired PFE & Diagnosed sarcopenia** |  |
| **Ideal lifestyle pattern** (87/1478) | | | | | | | 0.644 |
| Event/Total | 18/594 | 9/168 | 0/44 | 16/365 | 24/217 | 20/90 |  |
| Incidence rate | 3.39 (2.13–5.37) | 6.72 (3.14–11.58) | 0.00 | 4.92 (3.01–8.02) | 12.68 (8.50–18.92) | 26.18 (16.89–40.58) |  |
| Crude model | Reference | 1.72 (0.77–3.82) | - | 1.43 (0.73–2.80) | 3.55 (1.92–6.54) | 7.89 (4.18–14.92) |  |
| Model 1 | Reference | 1.69 (0.76‒3.78) | - | 1.06 (0.53‒2.11) | 2.14 (1.19‒4.23) | 2.82 (1.35‒5.90) |  |
| Model 2 | Reference | 1.80 (0.80–4.03) | - | 1.01 (0.50–1.99) | 2.37 (1.26–4.49) | 1.99 (0.88–4.48) |  |
| **Without ideal lifestyle pattern** (1449/10549) | | | | | | |  |
| Event/Total | 232/4102 | 114/1131 | 57/258 | 360/2606 | 318/1486 | 368/966 |  |
| Incidence rate | 6.39 (5.61–7.26) | 11.51 (9.58–13.83) | 26.46 (20.41–34.30) | 16.02 (14.45–17.76) | 25.54 (22.88–28.51) | 48.80 (44.06–54.05) |  |
| Crude model | Reference | 1.69 (1.28–2.23) | 4.17 (2.94–5.93) | 2.33 (1.89–2.89) | 4.20 (3.41–5.16) | 8.97 (7.33–10.97) |  |
| Model 1 | Reference | 1.48 (1.18‒1.85) | 2.17 (1.61‒2.92) | 1.72 (1.45‒2.03) | 2.19 (1.83‒2.62) | 2.87 (2.38‒3.46) |  |
| Model 2 | Reference | 1.52 (1.21–1.90) | 1.86 (1.37–2.53) | 1.67 (1.41–1.98) | 2.23 (1.86–2.67) | 2.48 (2.03–3.03) |  |

Ideal lifestyle pattern was defined as non-current drinkers, non-current-smokers and physically active.

Model 1: adjusted for sex (male or female), age (continuous), residence (urban or rural), education level (below lower secondary, upper secondary and vocational training, or tertiary education), and disease status (diabetes, hypertension, cardiovascular disease, arthritis, dyslipidemia and cancer).

Model 2: additionally adjusted for body mass index (continuous).

Abbreviations: CAPTURE, chronic obstructive pulmonary disease assessment in primary care to identify undiagnosed respiratory disease and exacerbation risk; CI, confidence interval; HR, hazard ratio; INTm, multiplicative interaction; PEF, peak expiratory flow.

# **Supplementary eTable 7.** Sensitivity analysis

|  | **Joint associations of sarcopenia and impaired PEF with all-cause death, HRs (95% CIs)** | | | | | | ***P*-trend** |
| --- | --- | --- | --- | --- | --- | --- | --- |
|  | **Normal PEF & Non-sarcopenia** | **Normal PEF & Possible sarcopenia** | **Normal PEF & Diagnosed sarcopenia** | **Impaired PEF & Non-sarcopenia** | **Impaired PEF & Possible sarcopenia** | **Impaired PFE & Diagnosed sarcopenia** |  |
| **Excluding death within one year of follow-up** | | | | | | | |
| Event/Total | 240/4686 | 117/1293 | 55/300 | 346/2941 | 321/1682 | 366/1034 |  |
| Incidence rate | 5.76 (5.08–6.54) | 10.27 (8.57–12.31) | 21.57 (16.56–28.10) | 13.46 (12.11–14.95) | 22.39 (20.07–24.98) | 44.12 (39.83–48.88) |  |
| Crude model | Reference | 1.76 (1.41–2.20) | 3.94 (2.94–5.28) | 2.35 (1.99–2.77) | 3.92 (3.32–4.63) | 8.13 (6.91–9.57) | <0.001 |
| Model 1 | Reference | 1.46 (1.17–1.83) | 2.04 (1.51–2.76) | 1.59 (1.34–1.88) | 2.15 (1.81–2.57) | 2.82 (2.34–3.40) | <0.001 |
| Model 2 | Reference | 1.51 (1.21–1.88) | 1.76 (1.29–2.40) | 1.55 (1.31–1.83) | 2.19 (1.84–2.62) | 2.45 (2.00–2.99) | <0.001 |
| **Excluding individuals with respiratory diseases** | | | | | | | |
| Event/Total | 222/4300 | 111/1179 | 48/268 | 254/2358 | 257/1337 | 260/785 |  |
| Incidence rate | 5.82 (5.10‒6.63) | 10.73 (8.91‒12.93) | 21.21 (15.99‒28.14) | 12.36 (10.93‒13.98) | 22.75 (20.14‒25.71) | 41.34 (36.61‒46.69) |  |
| Crude model | Reference | 1.83 (1.45–2.29) | 3.81 (2.79–5.21) | 2.14 (1.79–2.56) | 3.95 (3.30–4.73) | 7.43 (6.21–8.88) | <0.001 |
| Model 1 | Reference | 1.55 (1.23–1.95) | 1.92 (1.40–2.65) | 1.52 (1.27–1.83) | 2.25 (1.87–2.72) | 2.57 (2.09–3.16) | <0.001 |
| Model 2 | Reference | 1.58 (1.25–1.98) | 1.75 (1.25–2.43) | 1.50 (1.25–1.80) | 2.29 (1.89–2.76) | 2.35 (1.83–2.93) | <0.001 |
| **Excluding individuals with arthritis** | | | | | | | |
| Event/Total | 153/2945 | 63/722 | 31/155 | 227/1742 | 191/888 | 240/588 |  |
| Incidence rate | 5.86 (4.99‒6.86) | 9.92 (7.75‒12.70) | 23.91 (16.81‒34.01) | 15.05 (13.21‒17.14) | 25.95 (22.52‒29.91) | 53.14 (46.83‒60.31) |  |
| Crude model | Reference | 1.67 (1.25‒2.25) | 4.33 (2.95‒6.38) | 2.58 (2.10‒3.16) | 4.51 (3.64‒5.57) | 9.46 (7.73‒11.59) | <0.001 |
| Model 1 | Reference | 1.48 (1.10‒1.98) | 2.21 (1.49‒3.29) | 1.70 (1.37‒2.09) | 2.45 (1.96‒3.06) | 3.08 (2.43‒3.91) | <0.001 |
| Model 2 | Reference | 1.51 (1.12‒2.02) | 1.92 (1.28‒2.89) | 1.66 (1.34‒2.05) | 2.49 (1.99‒3.12) | 2.70 (2.09‒3.48) | <0.001 |

Model 1: adjusted for sex (male or female), age (continuous), residence (urban or rural), education level (below lower secondary, upper secondary and vocational training, or tertiary education), smoking habits (never, ever or current), alcohol consumption (yes or no), physical activity (active or inactive) and disease status (diabetes, hypertension, cardiovascular disease, arthritis, and dyslipidemia and cancer).

Model 2: additionally adjusted for body mass index (continuous).

The incidence rate indicates the number of deaths per 1000 person-years.

Abbreviations: CI, confidence interval; HR, hazard ratio; PEF, peak expiratory flow.

# **Supplementary eTable 8.** Association of all-cause death with joint exposure to sarcopenia and Chinese criteria-defined impaired respiratory function

|  | **Joint associations of sarcopenia and impaired PEF with all-cause death, HRs (95% CIs)** | | | | | | ***P*-trend** |
| --- | --- | --- | --- | --- | --- | --- | --- |
|  | **Normal PEF & Non-sarcopenia** | **Normal PEF & Possible sarcopenia** | **Normal PEF & Diagnosed sarcopenia** | **Impaired PEF & Non-sarcopenia** | **Impaired PEF & Possible sarcopenia** | **Impaired PFE & Diagnosed sarcopenia** |  |
| Event/Total | 243/3939 | 149/1136 | 111/388 | 383/3728 | 316/1866 | 334/970 |  |
| Incidence rate | 7.00 (6.15‒7.91) | 15.11 (12.87‒17.75) | 34.73 (28.84‒41.83) | 11.77 (10.65‒13.01) | 19.90 (17.81‒22.21) | 43.60 (39.17‒48.54) |  |
| Crude model | Reference | 2.16 (1.76‒2.65) | 5.22 (4.17‒6.53) | 1.69 (1.44‒1.99) | 2.85 (2.41‒3.37) | 6.58 (5.58‒7.76) | <0.001 |
| Model 1 | Reference | 1.58 (1.28‒1.94) | 1.93 (1.52‒2.44) | 1.63 (1.38‒1.91) | 2.09 (1.76‒2.47) | 2.80 (2.34‒3.34) | <0.001 |
| Model 2 | Reference | 1.64 (1.33‒2.02) | 1.67 (1.31‒2.13) | 1.61 (1.37‒1.89) | 2.15 (1.81‒2.55) | 2.42 (2.00‒2.93) | <0.001 |

Model 1: adjusted for sex (male or female), age (continuous), residence (urban or rural), education level (below lower secondary, upper secondary and vocational training, or tertiary education), smoking habits (never, ever or current), alcohol consumption (yes or no), physical activity (active or inactive) and disease status (diabetes, hypertension, cardiovascular disease, arthritis, and dyslipidemia and cancer).

Model 2: additionally adjusted for body mass index (continuous).

The incidence rate indicates the number of deaths per 1000 person-years.

Abbreviations: CI, confidence interval; HR, hazard ratio; PEF, peak expiratory flow.


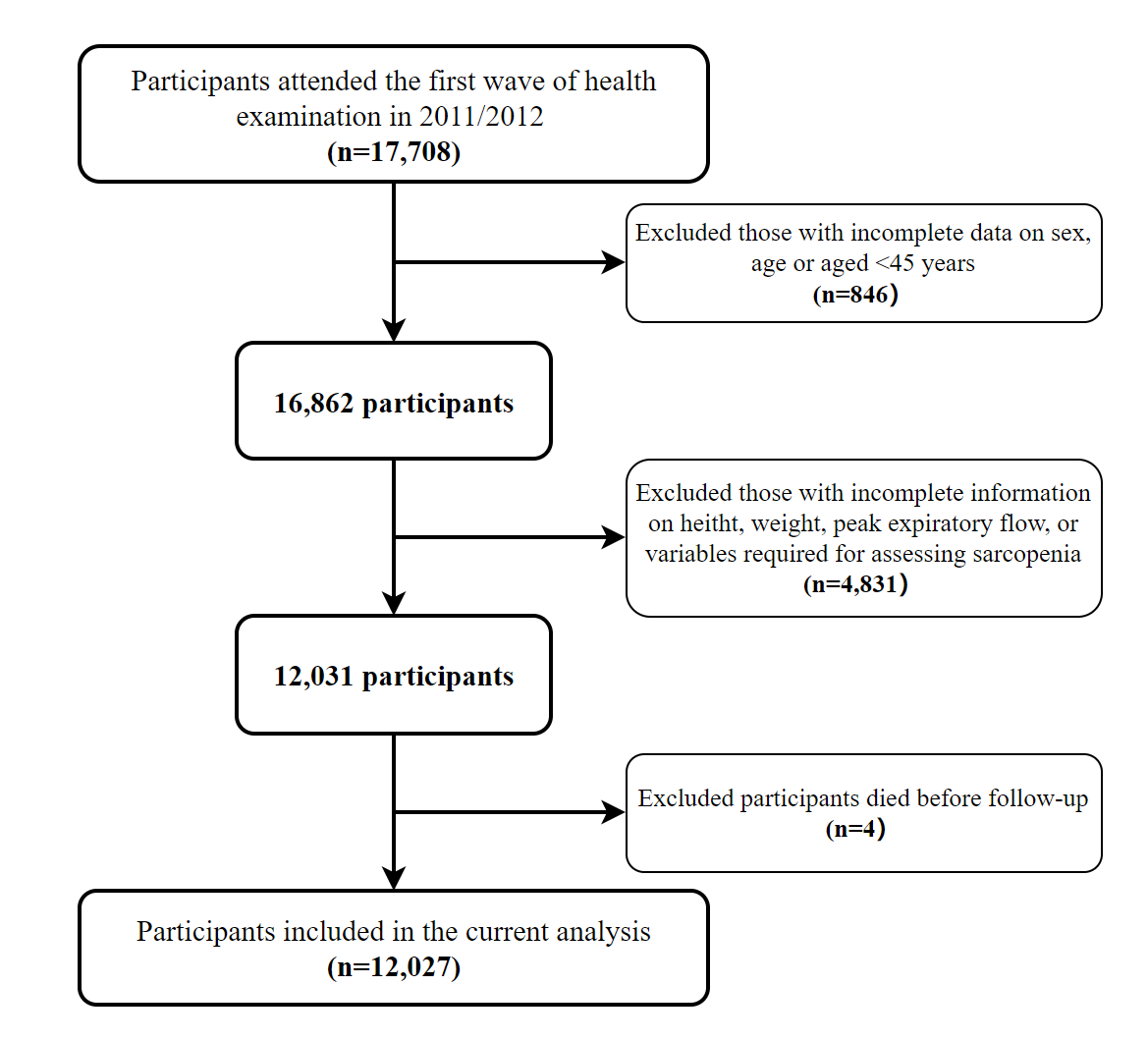


# **Supplementary eFigure 1.** Flowchart of the study population

**
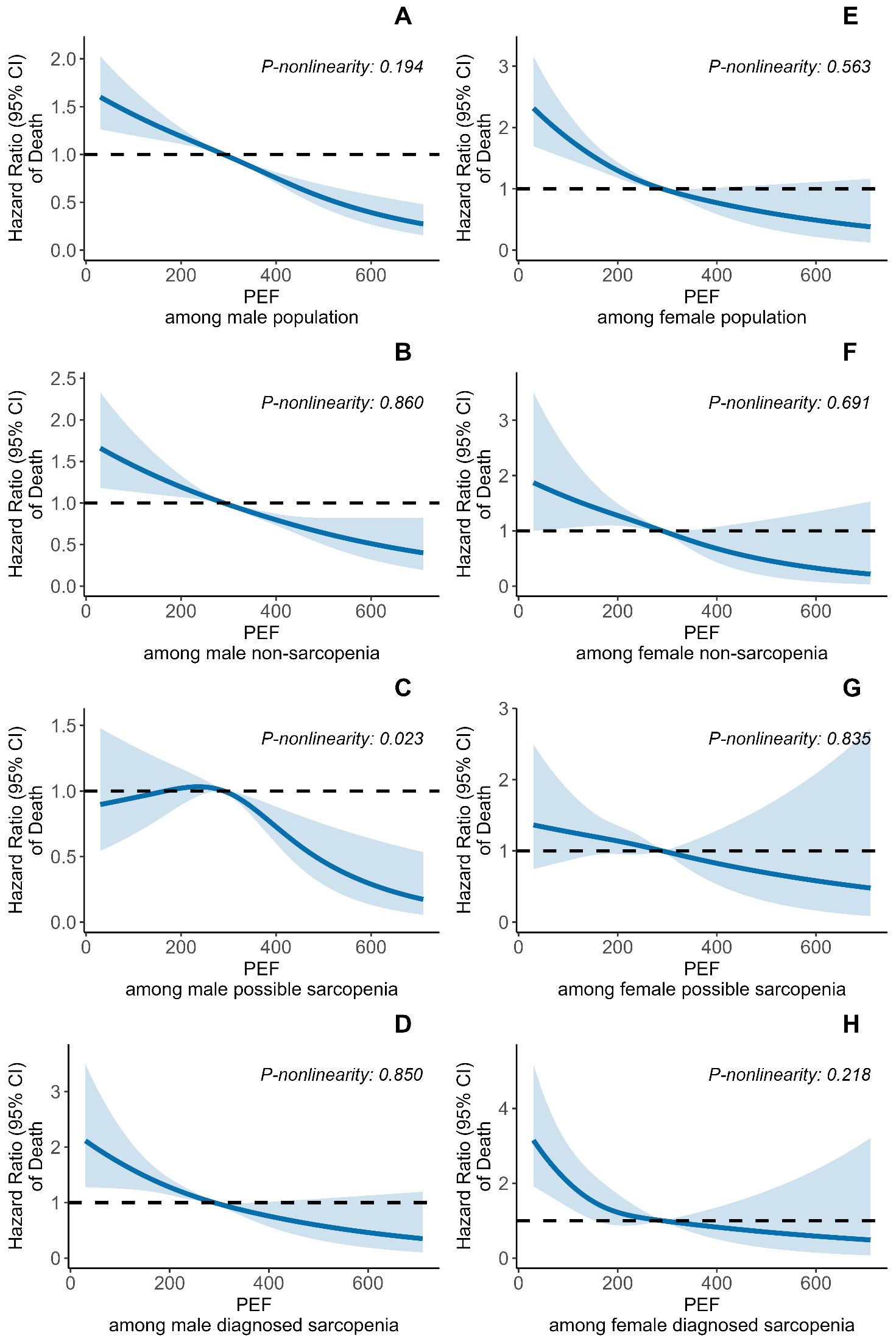
**

# **Supplementary eFigure 2.** Multivariable dose–response associations between peak expiratory flow (PEF) and all-cause mortality across sarcopenia subgroups stratified by and sex

**Caption**: Panels **A**–**D** show results for men; and Panels **E**–**H** for women.

Multivariable Cox models were adjusted for age (continuous), body mass index (continuous), residence (urban or rural), education level (below lower secondary, upper secondary and vocational training, or tertiary education), smoking status (never, ever, or current), alcohol consumption (yes or no), physical activity (active or inactive), and comorbidities (diabetes, hypertension, cardiovascular disease, arthritis, dyslipidemia, and cancer).

# **Supplementary eMethods**

**Assessment of sarcopenia**

Sarcopenia status was assessed according to the Asian Working Group for Sarcopenia (AWGS) 2019 criteria, which integrating the muscle mass, strength and physical performance into the assessment of sarcopenia[1]. Diagnosed sarcopenia is diagnosed when low muscle mass plus low muscle strength or low physical performance are detected. Possible sarcopenia is defined by low muscle strength or reduced physical performance.

The muscle mass was estimated by the appendicular skeletal muscle mass (ASM) using a previously validated equation in a Chinese population [2-4].


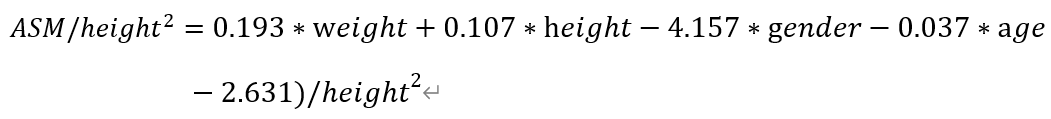


The cut-off for defining low muscle mass was set at the sex-specific lowest 20% of height-adjusted muscle mass (ASM/Height²) within the study population[4, 5]. This criterion has been well adopted in subsequent studies[2, 4-6]. For the current analysis, the sex-specific cutoff was <7.01 in men and <5.29 in women, consistent with the findings of previous study in the same population [5].

Muscle strength was indicated by grip strength. Handgrip strength was measured by a trained examiner using a YuejianTM WL-1000 dynamometer (Nantong Yuejian Physical Measurement Instrument Co., Ltd., Nantong, China) in kilograms. Subjects were in standing position and began the test using dominant or nondominant hand while receiving verbal encouragement. Every participant was measured twice for both left and right hand by holding the dynamometer at a right angle (90°) and squeezing the handle for a few seconds. Participants were asked to provide maximum effort for the measures. We used the maximum available values measured twice with left and right hand, and then took the average of maximum available values. If the participants unable to perform grip strength measurement in either hand due to health reasons (swelling, inflammation, severe pain, or injury), we would use the value measured by the other hand. According to the suggestion of AWGS 2019, the cut-off points for low handgrip strength are <18 kg in women and <28 kg in men[6].

Low physical performance was indicated by gait speed <1.0 cm/s or 5-time chair stand test ≥12s. Gait speed was assessed by measuring the participants’ usual gait (in m/s) in a 2.5-m course. According to the requirement, the participants walked the course at their usual pace twice (there and back). A stopwatch was used to time how fast the participant could walk. We took the average of available values twice. The 5-time chair rise time measures the amount of time needed for the participants to rise continuously five times keeping their arms folded across their chest from the height of the 47‐cm chair. We used the value of amount of time participants held stand in seconds.

**Assessment of peak expiratory flow**

The peak expiratory flow (PEF) was measured via a vital peak flow meter (Everpure ™, Shanghai, China), with the results recorded only if maximal effort was demonstrated. Each participant performed the maneuver three times at 30-second intervals under the guidance of trained technicians, and the highest value was used for analysis. Respiratory dysfunction was defined as per standard deviation (SD) decrease in PEF as a continuous form and using the COPD Assessment in Primary Care to Identify Undiagnosed Respiratory Disease and Exacerbation Risk (CAPTURE) criteria. A PEF <250 L/min for females and <350 L/min for males define impaired PEF[7].

In the sensitivity analysis, we redefined impaired PEF according to the Chinese criteria established by Nanshan Zhong *et al.*, where impaired PEF is defined as a PEF <80% of the predicted value (pPEF)[8]. Calculation algorithm for predicting peak expiratory flow in both sexes were as

$$pPEF\left( Male \right)=75.6+20.4*Age-0.41*{Age}^{2}+0.002*{Age}^{3}+1.19*Height$$

$$pPEF\left( Female \right)=282.0+1.79*Age-0.046*{Age}^{2}+0.68*Height$$

**Reference:**

1. Chen, L.K., et al., *Asian Working Group for Sarcopenia: 2019 Consensus Update on Sarcopenia Diagnosis and Treatment.* J Am Med Dir Assoc, 2020. **21**(3): p. 300-307.e2.

2. Gao, K., et al., *Association between sarcopenia and cardiovascular disease among middle-aged and older adults: Findings from the China health and retirement longitudinal study.* EClinicalMedicine, 2022. **44**: p. 101264.

3. Wen, X., et al., *Anthropometric equation for estimation of appendicular skeletal muscle mass in Chinese adults.* Asia Pac J Clin Nutr, 2011. **20**(4): p. 551-6.

4. Yang, M., et al., *Sarcopenia predicts readmission and mortality in elderly patients in acute care wards: a prospective study.* J Cachexia Sarcopenia Muscle, 2017. **8**(2): p. 251-258.

5. Qiu, W., et al., *Trend in prevalence, associated risk factors, and longitudinal outcomes of sarcopenia in China: A national cohort study.* J Intern Med, 2024. **296**(2): p. 156-167.

6. Wu, X., et al., *Sarcopenia prevalence and associated factors among older Chinese population: Findings from the China Health and Retirement Longitudinal Study.* PLoS One, 2021. **16**(3): p. e0247617.

7. Martinez, F.J., et al., *Discriminative Accuracy of the CAPTURE Tool for Identifying Chronic Obstructive Pulmonary Disease in US Primary Care Settings.* JAMA, 2023. **329**(6): p. 490-501.

8. 中华医学会呼吸病学分会肺功能专业组, *肺功能检查指南——呼气峰值流量及其变异率检查.* Chinese Journal of Tuberculosis and Respiratory Diseases 2017. **40**(06): p. 426-430.
